# Measuring and reporting quality of life outcomes in clinical trials in cystic fibrosis: a critical review

**DOI:** 10.1186/1477-7525-3-19

**Published:** 2005-03-24

**Authors:** Janice Abbott, Anna Hart

**Affiliations:** 1 Faculty of Health, University of Central Lancashire, Preston, PR1 2HE, UK

## Abstract

Good quality clinical trials are essential to inform the best cystic fibrosis (CF) management and care, by determining and comparing the effectiveness of new and existing therapies and drug delivery systems. The formal inclusion of quality of life (QoL) as an outcome measure in CF clinical trials is becoming more common. Both an appropriate QoL measure and sound methodology are required in order to draw valid inferences about treatments and QoL. A review was undertaken of randomised controlled trials in cystic fibrosis where QoL was measured. EMBASE, MEDLINE and ISI Web of Science were searched to locate all full papers in the English language reporting randomised controlled trials in cystic fibrosis, published between January 1991 and December 2004. All Cochrane reviews published before December 2004 were hand searched. Papers were included if the authors had reported that they had measured QoL or well being in the trial. 16 trials were identified. The interventions investigated were: antibiotics (4); home versus hospital administration of antibiotics (1); steroids (1); mucolytic therapies (6); exercise (3) and pancreatic enzymes (1). Not one trial evaluated in this review provided conclusive results concerning QoL. This review highlights many of the pitfalls of QoL measurement in CF clinical trials and provides constructive information concerning the design and reporting of trials measuring QoL.

## Review

### Cystic fibrosis and its management

Cystic fibrosis (CF) is a life threatening, recessively inherited disease caused by defects in a single gene on chromosome 7 [1,2]. The faulty gene causes an increased production of thickened secretions in most organs of the body. In the respiratory tract this impairs the clearance of micoorganisms resulting in recurrent infections, inflammation, lung damage and eventually death from respiratory failure. In the pancreas, the pancreatic exocrine cells become blocked, leading to the failure of the pancreas to produce digestive enzymes causing the maldigestion and malabsorption of nutrients. Current median survival is more than 30 years [3,4], and half of children born in the 1990's are expected to survive to more than 40 years [5]. With increasing age, however, a high proportion of people develop diabetes mellitus, and some patients endure a variety of complications including pneumothorax, haemoptysis, chronic liver disease and osteoporosis.

Cystic fibrosis has an extraordinarily demanding treatment regimen. The management of respiratory disease is directed at identifying and eradicating bacterial infection from the airways. Preventing chronic infection with *Pseudomonas aeruginosa *can slow down the deterioration in lung function and improve survival [6,7]. Respiratory disease is treated with antibiotics, mucolytics, bronchodilators and corticosteroids. Additionally, twice daily chest physiotherapy and an exercise regimen aid the clearance of respiratory secretions. The management of gastrointestinal symptoms hinges on the maintenance of body weight and, therefore, a high energy, high fat diet is prescribed. To aid the absorption of nutrients, oral pancreatic enzymes are taken with food. Malnutrition is managed with fat-soluble vitamins and oral feed supplements and/or where required, nocturnal enteral feeding. CF-related diabetes requires insulin, and further therapies are required to deal with other complications.

### The importance of clinical trials in CF

Effective treatments, in particular those that are acceptable to patients, are crucial to slow down disease progression. Good quality clinical trials are essential to generate evidence to inform CF management, by determining and comparing the effectiveness of therapies and drug delivery systems. Often, the ultimate aim of treatment is to increase survival, but it is impractical for trials involving younger patients to have survival as a primary end-point. Instead investigators opt for proxy outcome measures. The progression of CF disease is usually evaluated by changes in lung function; forced expiratory volume in 1 second (FEV_1_) being the typical primary outcome measure. It has been commonplace for researchers to conclude that because treatments had benefits for clinical outcomes there must also have been benefits for QoL. There are many such assertions in the literature without empirical evidence, however, the formal inclusion of QoL as an outcome measure in CF clinical trials is now increasing.

### The quality of clinical trials

There has been considerable concern about the quality of reporting of randomised controlled trials (RCTs). Poorly-designed trials are likely to produce biased results and hence misinformed decision-making. Inadequate reporting means that readers are unable to assess the quality of the design of an RCT and are consequently unable to assess the usefulness of the conclusions. The CONSORT statement [8] was devised to improve the quality of reporting of RCTs. Both an appropriate QoL measure and sound methodology are required for valid inferences about treatments and QoL. With this in mind a review was undertaken of RCTs in cystic fibrosis where QoL was measured.

### The identification of papers

EMBASE, MEDLINE and ISI Web of Science were searched to locate all full papers in the English language reporting RCTs in cystic fibrosis, published between January 1991 and December 2004. Papers were located that included 'cystic fibrosis' and 'quality of life' or 'health status' in the title, abstract or keywords, and these were hand-searched for all randomised controlled trials. Extra searches on 'well-being' were carried out in databases where this key word was accepted. Papers were included if authors reported that they had measured QoL or well-being. All Cochrane reviews [9] published before December 2004 were hand-searched. Only trials exclusively on people with cystic fibrosis were included i.e. trials were omitted where some of the subjects had cystic fibrosis. Papers were omitted which described a study on a subset of subjects in an RCT, but secondary analyses of RCTs where the primary findings had previously been published were included. Each author undertook searches and reviews independently. A summary sheet was used to record the following: rationale for measuring QoL; choice of QoL instrument, quality of scale description and scoring methods; sample size justification; quality of summary statistics for QoL and analysis; discussion of the clinical importance of QoL findings and general methodological quality in relation to QoL

A total of 16 trials were identified. The interventions investigated were: antibiotics (4); home versus hospital administration of antibiotics (1); steroids (1); mucolytic therapies (6); exercise (3); and pancreatic enzymes (1). The trials, with patient characteristics, are summarised in Tables 1 and 2. Table 1 presents a summary of RCTs evaluating antibiotics and steroids. Table 2 summarises the RCTs evaluating mucolytic therapies, exercise and pancreatic enzymes.

**Table 1:** Summary of RCTs in CF measuring QoL: antibiotics and steroids

**Authors**	**Brief description**	**Sample**	**QoL scale used**	**Authors' main conclusion**	**Authors' conclusion about QoL outcome**
**ANTIBIOTICS**					
Quittner et al. [14] Secondary analysis of Ramsey et al. [13]	Secondary analysis of RCT. Tobramycin versus placebo in 3 cycles	n = 520 (age >= 6 years) (n = 499 for QoL) mean age 21 years FEV_1 _25% to 75%	Non-validated 3-point scale (better/no change/worse) primary outcome	Tobramaycin improved lung function (not reported here)	Tobramycin associated with improved QoL
Equi et al. [17]	Azithromycin (250 gm or 500 gm dependent on weight) vs placebo; crossover design	n = 41 (age 8–18 years) median FEV_1 _= 61% (range 33% to 80%)	QWB	Significant improvement in FEV_1 _compared with placebo, but not FVC or mid-expiratory flow	No difference in QoL
Wolter et al. [18]	Azithromycin vs placebo; 2 parallel groups	n = 59 (age 18–44 years; mean 27.9 years) mean FEV_1 _= 56.5%	CRQ	Significant difference in FEV_1 _and FVC favouring azythromycin	Significant improvement in all domains of QoL
Saiman et al. [19]	Azithromicyn vs placebo; 2 parallel groups	n = 185 (age >= 6 years) (n= 177 for QoL) mean age 20 years ≈ 60% had FEV_1 _> 60%	CFQ	Significant difference in FEV_1_, lower risk of exacerbation, higher weight, but more side effects in treatment group	Significant difference in physical functioning domain only
**HOME/HOSPITAL ANTIBIOTICS**					
Wolter et al. [20]	Home versus hospital IVs antibiotics; 2 parallel groups	17 adolescents and adults	CRQ primary outcome	No clinical compromise associated with home therapy	Home IVs fared worse for fatigue and mastery, but better for personal, family, sleeping, eating and total disruption
**STEROIDS**					
Balfour-Lynn et al. [24]	Corticosteroids vs placebo; crossover	n = 22 (age 7–17 years; mean 10.3 years) mean FEV_1_= 64% (range 21% to 102%)	Ad hoc VAS scales	No significant benefit in any of the outcomes	No changes in well-being

FEV_1 _= forced expiratory volume in one second, expressed as percent predicted; FVC = forced vital capacity; QWB = Quality of Well-being Scale; CFQ = Cystic Fibrosis Questionnaire; CRQ = Chronic Disease Respiratory Questionnaire; ≈ = approximately. QoL is secondary outcome measure unless otherwise indicated. All authors refer to QoL in the title, abstract or paper except [24] who refer to well-being.

**Table 2:** Summary of RCTs in CF measuring QoL: mucolytic therapies, exercise and pancreatic enzymes

**Authors**	**Brief description**	**Sample**	**QoL scale used**	**Authors' main conclusion**	**Authors' conclusion about QoL outcome**
**MUCOLYTIC THERAPIES**					
Ranasinha et al. [27]	DNase vs placebo; two parallel groups	n = 71 (age = 16–55 years) mean FEV_1 _≈ 47%	Ad hoc 9-item scale	Significant improvement in FEV_1 _but not in FVC	DNase did not improve overall well-being but improvements in feeling, cough frequency and chest congestion
Ramsey et al. [28]	3 doses of DNase vs placebo; 4 parallel groups	n = 181 (age 8–65 years) mean FEV_1 _between 58.6% and 84.6% for the 4 groups	Ad hoc 9-item scale	FEV_1 _and FVC improved across all doses compared with placebo	DNase associated with decreased dyspnoea and improved well-being
Fuchs et al. [29]	2 doses of DNase vs placebo; 3 parallel groups	n= 968 (age 5–54 years) mean FEV_1 _≈ 60%	Ad hoc 9-item scale	Improved lung function on DNase	Increase in general well-being
Wilmott et al. [30]	2.5 mg DNase or placebo twice daily	n = 80 children and adults (age >5 years; mean ≈ 20) mean FEV_1 _≈ 40%	Ad hoc scale	No effect of drug on change in FEV_1 _or FVC	No differences on well-being scales
Suri et al. [31-33]	Open crossover study of DNase once daily 2.5 mg vs alternate day 2.5 mg and saline	n = 48 (age 7–17 years) (n = 40 completed study)	QWB-SA	No evidence of differences between active treatments; daily treatment better than saline for FEV_1_	No effects
Eng et al. [34]	10 ml of either normal or hypertonic saline; parallel groups	n = 58 (age 7–26 years) mean FEV_1_≈ 52%	Ad hoc VAS of perceived change	Significant differential improvement from baseline in FEV_1 _for hypertonic saline	An improvement, but group difference did not reach statistical significance
**EXERCISE**					
Selvadurai et al. [36]	Comparison of aerobic/ resistance training and standard care; 3 parallel groups	n = 66 (age 8–16 years) mean FEV_1 _≈ 57%	QWB	Aerobic training better for peak aerobic capacity. Resistance training better for weight gain, lung function and leg strength	Aerobic training associated with better QoL
Klijn et al. [37]	Anaerobic training vs normal daily activity; 2 parallel groups	n = 20 (age 9–18 years; mean 14 years) mean FEV_1 _= 75.2% (exercise group); 82.1% (control group)	Dutch CFQ	Anaerobic and aerobic performance improved in training group, but not control group	QoL improved in training group but not in control group
Orenstein et al. [38]	Aerobic versus upper-body strength training	n = 62 (age 8–18 years) Analysis on 53 cases of complete data	QWB	Strength and aerobic training may increase upper-body strength, and physical work capacity	No significant effects
**PANCREATIC ENZYMES**					
Gan et al. [39]	4 versus 1 capsule daily crossover design	n = 13 (age 19–46 years; mean 28 years) mean BMI = 21	Symptoms and general well-being on 10-point scale	No difference between treatments	No significant changes in scores for well-being

FEV_1 _= forced expiratory volume in one second, expressed as percent predicted; FVC = forced vital capacity; QWB = Quality of Well-being Scale; CFQ = Cystic Fibrosis Questionnaire; BMI= body mass index, ≈ = approximately. QoL is secondary outcome measure unless otherwise indicated. All authors refer to QoL in the title, abstract or paper except [34] [39] who refer to well-being.

## Review of CF trials that have measured QoL

### Antibiotic therapy

One of the most important therapies for CF is antibiotics aimed at preventing, eradicating and controlling respiratory infection. *Pseudomonas aeruginosa (PA) is *the most prevalent infection [10], and the prognosis of chronically infected patients is considerably worse [11]. It is also known that pulmonary exacerbations have a strong negative impact on QoL in CF [12], therefore an effective antibiotic should be able to demonstrate improvements in QoL. Of the four antibiotic trials that reported measuring QoL in CF (as a secondary outcome) one evaluated tobramycin and the other three assessed azythromycin.

Tobramycin's mode of delivery is appealing to patients as it is nebulised, delivering high concentrations of the drug to the site of infection; making the treatment less complex and time-consuming than by intravenous administration. Ramsey et al. [13] conducted a placebo-controlled RCT of inhaled tobramycin and concluded that the drug improved lung function. Quittner et al. [14] reported on QoL from a secondary analysis of this work. Patients were assigned to receive either tobramycin (300 mg twice daily) or placebo for three treatment cycles, with each cycle consisting of 28 days on the drug followed by 28 days off the drug. QoL was assessed only at the end of each treatment period (28 days on the drug) using a non-validated, three point, uni-dimensional global rate of change questionnaire. Patients (or parents of some children) and physicians reported whether the patient's condition remained unchanged, improved or deteriorated. The authors reported that a greater number of patients receiving tobramycin felt better at the end of each treatment cycle. There is evidence that patients taking the drug were more likely to report an improvement in their condition after each period of treatment. However, how 'feeling better or worse' would impact on different aspects of a person's QoL is unknown and the authors themselves suggest that evaluation with CF-specific QoL scales would be beneficial. It is also unclear how many children's ratings or parental proxy ratings were included in the analysis. Pre-treatment data were not collected so it is unknown what happened in the periods off treatment. Without this information, and the magnitude of all changes, it is impossible to deduce what the overall changes would have been if QoL had been measured at baseline and at 6 months.

Macrolide antibiotics are unable to kill *PA *but it is thought that they may reduce the activity of the bacteria [15]. Azythromycin has become a focus of interest in CF because it is possible that it reduces sputum viscosity and airway adhesion of *PA *[16]. If a drug can reduce bacterial activity and inflammation, and enable sputum to be expectorated more easily, it may be expected to improve QoL. If it can also be administered orally, rather than being given intravenously or via a nebuliser, its potential impact on QoL may be considerable. Equi et al. [17] undertook a 15 month, randomised, double blind, placebo-controlled crossover trial. Patients received either azythromycin (bodyweight <40 kg = 250 mg daily; >40 kg = 500 mg daily) or placebo for six months. This was followed by a two month washout period prior to the treatments being crossed over. Lung function was the primary outcome measure. QoL was one of several secondary outcomes and was measured using the Quality of Well Being Scale (QWB). The scale is not described in the paper but it is a utility instrument, typically administered by interview. It generates a single score by summing the scores of the three subscales: mobility, physical activity and social activity. The authors reported an improvement in FEV_1 _for the drug compared with placebo. The only results given for QoL were: 'The median difference in the visual analogue score (range 0–100) for well being between the end of the azythromycin and placebo treatment periods was 0, as was the change in the total quality of well being score'. It cannot be ascertained whether the use of a CF specific scale would have detected treatment differences.

Wolter et al. [18] reported data from a parallel design RCT comparing azythromycin with placebo. Patients took azythromycin 250 mg/day or placebo for three months and were assessed at baseline and each subsequent month for lung function, weight and QoL. Quality of life was measured using the Chronic Respiratory Disease Questionnaire (CRQ). The CRQ has four subscales: fatigue, mastery, emotion and dyspnoea which can be summed to provide a total QoL score. The authors reported that treatment with azythromycin significantly reduced the rate of decline in lung function over time. Improvements in the domains of mastery, emotion, dyspnoea and total score were greater for those who received azythromycin. Improved fatigue scores were only seen in the azythromycin group. Although means and standard deviations of QoL scores are presented for each treatment group, there are a high proportion of missing values, and in the absence of confidence intervals it is difficult to assess the clinical importance of the results.

Similarly, Saiman et al. [19] described a parallel designed, placebo-controlled RCT to 'determine any association between azythromycin and lung function in CF'. Treatment was prescribed (bodyweight <40 kg = 250 mg; >40 kg = 500 mg) on three days each week for six months. QoL was one of several secondary outcome measures, and was evaluated using the USA Cystic Fibrosis Questionnaire (CFQ). Unfortunately, the scale, which is a CF specific QoL measure, was not described in the paper. The CFQ child measure is a 33 item self report instrument that includes three broad domains of QoL: physical symptoms, emotional functioning and social functioning and there are five domains that are specific to CF; body image, eating disturbances, treatment burden, respiratory symptoms and digestive symptoms. The adult version has 48 Items across 12 domains: physical, role, vitality, emotion, social, body, eating, treatment, health, weight, respiratory and digestion. The authors presented the QoL results as 'three broad factors' (physical, psychosocial, and body image) plus a total score. Presumably, these factors were an amalgamation of domains, although no explanation is provided as to how they were derived. The authors also appear to have combined the child and adult versions of the CFQ but provide no rationale or account of how this was done. A differential improvement in pulmonary function and nutritional status was reported for the azythromycin group, but for QoL the only statistically significant difference reported was for the 'physical factor'. The CFQ scales are usually scored from 0–100. If this is the case, the observed difference of 2.7 in the 'physical factor' change score is unlikely to have clinical importance for the patient. Even a difference of 5.3, which is the extreme of the stated 95% confidence interval, is likely to be only marginally clinically important. Moreover, the small difference reported can be largely accounted for by an average deterioration of 1.9 in the placebo group. The problems of interpretation are exacerbated by a lack of baseline data on QoL scores.

### Home intravenous antibiotic therapy

Home intravenous (IV) therapy is a popular form of treatment. It has the advantages of cost-saving by freeing up hospital beds and avoiding cross infection; and the patient and family are able to continue their normal activities. However, a crucial question is whether patients would adhere to their treatment sufficiently to ensure that the home and hospital environments would provide equivalent outcomes. Wolter et al. [20] conducted a parallel designed RCT of home compared with hospital IVs. The aim was 'to determine the equivalence of home and hospital care... so that if no difference was detected between the two modes of treatment, this could be stated with confidence'. Those randomised to home therapy spent 2–4 days in hospital being taught how to prepare and administer their own antibiotics. Data were obtained from 17 adolescent and adult patients for 31 admissions of respiratory exacerbation. Therapy consisted of ceftazidime, 2 g 12 hourly and tobramycin 4–6 mg/kg daily as a single bolus. QoL was the primary outcome, measured using the Chronic Respiratory Disease Questionnaire (CRQ). Patients were assessed on days 0, 10 (cessation of drug) and 21. On day 21 they were asked to score the degree of disruption to family, personal, sleeping and eating aspects of their life on a 7-point scale. The median duration of treatments was similar for both home and hospital groups. The authors reported no differences concerning dyspnoea and emotional functioning, but the fatigue, mastery and total CRQ scores were poorer for home patients. However, improved QoL was reported in the areas of personal, family, sleeping, eating and total disruption for home, compared with hospital admissions. There were no reported statistical differences in clinical outcome. QoL scores were the main outcome, but the study was only powered on the dyspnoea QoL scale. The sample size calculation, carried out during the study design, was based on 95% power to detect 'differences of 5 or more units in the dyspnoea score'. The authors reported that 'a 5 units difference... was hypothesised as an important change'. Yet after the data had been collected it was clear that the power calculation was invalid, and the sample too small. The estimated difference in dyspnoea change scores was 2.5, but no confidence intervals were presented. It is likely that a 95% confidence interval for this difference would contain values greater than 5.

### Anti-inflammatory therapy

Lung inflammation can occur very early in life [21] and corticosteroids have the potential to reduce lung damage arising from inflammation. These drugs are among the most potent anti-inflammatory agents available and there is widespread prescribing of inhaled steroids in CF [22]. Oral corticosteroids are associated with several adverse effects, although the adverse effects of inhaled steroids are fewer. However, a Canadian trial was stopped prematurely because of the increased frequency of *PA *[23]. Because there are perceived and potential benefits and harms of steroid treatment, QoL measurement is very useful. One such trial was located. Balfour-Lynn et al. [24] conducted a double blind, placebo-controlled, randomised crossover trial in which fluticasone propionate (400 ug daily) was given as a dry powder inhaler. The drug was inhaled for six weeks with a four-week washout period before crossover. There were several biochemical and clinical outcome measures. At the beginning and end of each treatment period, scores for general well-being and appetite were recorded on a 10 cm visual analogue scale, in order to establish symptom severity. The authors concluded that there were no changes in respiratory symptoms, well-being or appetite scores. However, there is no discussion of the clinical importance of the confidence intervals in what the authors acknowledge is a small and possibly underpowered study.

### Mucolytic therapies

Mucolytic agents enable respiratory secretions to be expectorated more easily. Dornase alfa (DNase) is a nebulised treatment intended for administration prior to chest physiotherapy to maximise chest clearance. Nebulised hypertonic saline is also a potential mucolytic therapy for CF. This review identified six RCTs of mucolytic therapies that measured QoL as a secondary outcome. Four of these were placebo-controlled trials of DNase, one compared DNase to hypertonic saline and one was a placebo-controlled trial of hypertonic saline.

Early studies evaluating the effective dosage, biochemical efficacy and safety of DNase in CF adults, employed a non-validated ad hoc measure of QoL [25,26]. This comprised five questions concerning general well-being (feeling, energy, physical activity, appetite, sleep) and four CF-related symptoms (cough frequency, cough severity, ease of sputum expectoration and chest congestion). Patients reported these items on a five-point Likert response scale. Additionally, the magnitude of dyspnoea was rated on a visual analogue scale. Even though there were no data describing the validity, reliability or sensitivity of the measure, it was subsequently used in several studies of DNase. These included the four randomised controlled trials of DNase reported here.

Ranasinha et al. [27] conducted a double-blind, placebo-controlled RCT in which patients received either 2.5 mg DNase twice daily or placebo for 10 days. The authors reported an improvement in lung function following DNase therapy. QoL was measured on seven occasions over the study period of 47 days (including pre-trial and follow-up). Only baseline QoL scores are reported but the authors infer that although there was no improvement in overall well-being or dyspnoea there were improvements in 'feelings', cough frequency and chest congestion. The total absence of any QoL change data makes it impossible for the reader to judge or interpret.

In a double blind placebo-controlled RCT performed by Ramsey et al. [28] children and adults were recruited. The trial consisted of four parallel groups (three different doses of DNase and placebo). The study period was 42 days with medication administered for the first ten days.Mean percentage change in lung function from baseline for each treatment group were provided at days 3, 10, 21 and 42. An improvement in lung function was observed during the administration of DNase, with values declining towards baseline following the treatment period. The authors reported a decreased perception of dyspnoea and an improved perception of well-being in the DNase groups compared to controls. Voice alteration and sore throat were more frequent among patients receiving DNase. However, QoL was not measured past day 10 so it is unclear whether QoL would have demonstrated similar trends to those of lung function. No baseline values are presented for QoL, but mean changes (from baseline to Day 10) are given for each group, without any measures of variability. There is no discussion of any possible dose-response (as is presented for FEV_1_), or of the clinical importance of the observed differences. These differences appear to be too small to be clinically important, and it is difficult to detect any consistent trends in the dose-response. Problems in interpretation are exacerbated by the lack of baseline data on QoL. It is impossible to judge if patients had scope for improvement, whether the scales were sensitive, or whether any particular dose is to be preferred with respect to QoL.

Fuchs et al. [29] conducted a double-blind, placebo-controlled RCT with three parallel groups (2.5 mg DNase once or twice daily and placebo). Children and adults were treated over a six-month period. Both doses of DNase resulted in slightly improved lung function. The authors report an increase in general well-being and a decrease in CF symptoms, although information is not available concerning specific symptoms of the measure (e.g. cough frequency). This was a large study and although the authors reported statistically significant differences these do not appear to be clinically important. There were ceiling effects in QoL measures at baseline: for each group the mean score was 3.9, with a maximum score of 5 (ceiling). Although the authors reported that the average change in well-being score for the once-daily group was significantly greater than for placebo, this change is extremely small, and is not replicated in the twice-daily group. There is therefore little convincing evidence about the effects of the treatments on QoL.

Wilmott et al. [30] undertook a double blind, placebo-controlled RCT with two parallel groups. Patients received either 2.5 mg DNase or placebo twice per day for 14 days. Clinical and QoL data were recorded on days 1, 8 and 15. Similar changes in lung function occurred in both groups. The authors reported no differences between the groups for any of the well-being or CF symptom items, but they do not provide enough information to enable the reader to interpret the data. There was evidence of greater improvement in dyspnoea (measured on the VAS) in the treatment group. This was statistically significant at day 8, but not at day 15, although the difference may have still been clinically important at day 15. This possible treatment effect was not reported in the QoL results, where, for all scales (including dyspnoea at different levels of activity) they report 'no difference between the groups'.

DNase is a comparatively expensive treatment for CF and not all patients can benefit from it. Hypertonic saline is an inexpensive potential alternative. A comparison of DNase and hypertonic saline was undertaken by Suri et al [[31-33] – publications of same trial]. They conducted an open, randomised crossover trial. Children were randomised to once daily DNase (2.5 mg), alternate day DNase (2.5 mg) or twice daily 5 mL 7% hypertonic saline in blocks of 12 weeks' duration with a two week washout period between treatments. In addition to the efficacy of the three treatments the study aimed to compare cost-effectiveness and therefore a utility measure of QoL, the Quality of Well-being Scale-Self Administered (QWB-SA), was chosen. The QWB-SA contains five domains: acute and chronic generic symptoms, self care, mobility, physical activity and performance of usual activities. These domains are combined to produce a well-being score of between 0 (death) and 1 (symptom-free full function). Parent and child completed the questionnaire together. The authors reported no difference in improved lung function between daily and alternate day DNase (16% and 14% improvement in FEV_1_% respectively). A mean FEV_1_% predicted improvement of only 3% was observed for the hypertonic saline condition and a statistically significant difference was reported between daily DNase and hypertonic saline. There were no significant changes from baseline for any treatment on the QWB-SA scale; neither were there differences between the treatment change scores. The trial was not powered for QoL and in one paper [31] the authors included confidence intervals. An improvement in reported well-being may be expected to accompany these relatively large changes in lung function in the DNase groups, yet the authors do not discuss possible reasons for these negative findings.

Eng et al. [34] conducted an open-label, placebo-controlled, parallel trial. Patients were randomly allocated to receive 10 ml of either normal saline (0.9% NaCl) or hypertonic saline (6.0%NaCl) twice daily (nebulised prior to physiotherapy) for two weeks. Change in lung function was the primary outcome and patient-perceived CF related symptoms were a secondary outcome. Patients rated perceived change of dyspnoea, fatigue, appetite, exercise tolerance, sleep and general well-being on a 10 cm visual analogue scale (VAS) on days 14 and 28. They also rated the effectiveness of sputum clearance on a similar VAS based on their diary information. Hypertonic saline improved lung function (at day 14) compared with normal saline; the mean increase in FEV_1_% predicted from baseline was 15% for the hypertonic saline compared with 2.8% for normal saline. The authors reported that the administration of hypertonic saline significantly improved exercise and the quality of sleep. For the other symptoms and general well-being there were no statistically significant differences between the groups. However, it is unlikely that the study was adequately powered for symptoms and well-being scores. While some of the estimated differences may only appear marginally important, it is probable the 95% confidence intervals for most of the symptoms and well-being scores would contain clinically important values. Given this problem, together with a total lack of information about QoL and symptoms at baseline, the QoL results are inconclusive.

### Exercise

Lung function and exercise tolerance decrease as CF disease progresses. Exercise training aims to preserve and improve fitness levels and enable CF patients to perform everyday activities. Patients with high levels of aerobic fitness have better survival than those with low fitness levels [35]. Aerobic training aims to improve cardiovascular function whereas anaerobic or resistance training aims to improve muscle strength. Three RCTs of exercise measuring QoL as a secondary outcome were located.

Selvadurai et al. [36] conducted an RCT to compare aerobic and resistance training. Children, admitted to hospital for a pulmonary exacerbation, were randomised to one of three parallel groups; aerobic training, resistance training and a control group. The mean duration of hospitalisation was around 19 days for each group. The exercise groups received five training sessions each week. All groups received in-patient standard care – intravenous antibiotics, chest physiotherapy and nutritional supplements. Peak aerobic capacity and lung function appear to be primary outcomes, measured on admission to hospital and at discharge. Quality of life, measured by the QWB scale, was assessed at baseline and one month following discharge. The authors do not describe the scale, its scoring method, or what the tabulated values represent. They report that aerobic training was associated with better peak aerobic capacity, activity levels and QoL, whereas children who received resistance training had better weight gain, lung function and leg strength.

Klijn et al. [37] investigated the effects of anaerobic training in non-hospitalised children who were randomly assigned to either a training group or control group. The exercise group trained for 30–45 minutes, twice each week for twelve weeks. There were several clinical and physiological outcomes. QoL was measured by the Dutch version of the Cystic Fibrosis Questionnaire (CFQ). A 47 item teen/adult scale and a 35 item child scale of the CFQ were employed but there was no description of the psychometric properties of this recently-translated scale. The domains of the scales were not described and neither was the instrument's scoring system. All outcome measures were evaluated at baseline, at the end of the training period and at 12 weeks' follow-up. The authors reported that by the end of the training period the children in the exercise group had improved their anaerobic and aerobic performance and QoL. At twelve weeks follow-up anaerobic performance and QoL remained higher than pre-training values. However, for QoL scores there are no direct statistical tests comparing the groups, and no confidence intervals. It also appears that data from the CFQ child and teen/adult scales were incorporated into the same analysis and it is unclear how this was achieved. There was inadequate reporting of summary statistics to enable the reader to interpret the findings, and selective reporting of QoL data. The CFQ scales consist of numerous domains but only the physical functioning domain was statistically significant. Even so, the authors conclude that 'anaerobic training has measurable effects on QoL'. Furthermore, the interpretation of the physical function data is unreliable because baseline imbalances in this score were not considered (training group mean 70.3; control group mean 83.2).

Orenstein et al. [38] conducted a one-year randomised trial to compare the effects of a home-based, semi-supervised, upper-body strength training regimen with a similarly structured aerobic training regimen. Sixty-two children participated in the trial although analysis was undertaken on 53 completed cases. Participants in both exercise conditions were visited at home once per week for the first eight weeks then monthly for the remainder of the study. All patients were encouraged to exercise at least three times per week for a year. Aerobic fitness, pulmonary function, strength and QoL were measured at baseline, 6 and 12 months. QoL was measured using the interview format of the QWB scale: for children younger than 12 years a parent responded. The authors concluded that strength and aerobic training may increase upper body strength and that both types of training may increase physical work capacity. There were no statistically significant differences for either within or between group analyses for QoL. Interpretation of results rests on p-values, with no discussion of clinical importance, although the reported differences in total well-being score appear to be too small to be of clinical importance.

### Pancreatic enzyme therapy

The majority of CF patients develop pancreatic insufficiency which leads to the maldigestion of dietary lipid, protein and carbohydrate. Pancreatic enzyme replacement therapy is used to manage maldigestion with patients taking numerous capsules each day. Gan et al. [39] conducted a double-blind, randomised crossover trial to compare a high lipase pancreatic enzyme preparation (Pancrease-HL) with regular Pancrease capsules. The rationale was that if the same strength of pancrease could be put into 1 capsule instead of 4, adherence to treatment may improve. Thirteen adults participated in two study periods of 14 days. During each period patients took 5 capsules 3 times per day; either 4 of Pancrease and 1 of placebo or 1 of Pancrease-HL and 4 placebo. The primary outcome measures appear to be mean fat and nitrogen excretion. General well-being was measured daily on a ten point scale. The authors reported no differences between the groups for any outcome. It is unclear whether the scores presented are baseline or change scores, or if the daily ratings were combined in some way to calculate the well-being scores. The authors' conclusion is that one capsule of the new preparation appears to be 'equivalent' to four capsules of the regular preparation, which suggests that the design should have been an equivalence study. However, in the absence of a clear hypothesis for the study, or a sample size calculation it is difficult to be sure. Whatever the hypothesis, the possible lack of power means that confidence intervals are essential for valid interpretation of results.

## Discussion of QoL data from CF trials

The value of the evidence from a trial depends on the quality of its design. Much of the data presented here was difficult to interpret. There are several reasons for this, and these are presented in this discussion.

### Choice of QoL measure

Only a few papers provided a rationale for measuring QoL [14,20,30,31] or a rationale for the choice of QoL instrument [18,20,30,31]. A variety of scales, reported to measure QoL, were employed: a uni-dimensional global rate of change questionnaire [14],'ad hoc' CF rating scales [24,27-30,34,39], the Quality of Well-being Scale [17,31,36,38], the Chronic Respiratory Disease Questionnaire [18,20], and the American [19] and Dutch [37] versions of the Cystic Fibrosis Questionnaire. The fact that a scale has been previously used on a CF population does not mean that it has been appropriately validated for use in CF studies. Apart from CF specific QoL instruments [40,41] only the SF-36 [42,43] and the Chronic Respiratory Disease Questionnaire (CRQ) [44] have undergone appropriate psychometric testing in CF. A chosen scale should be relevant to the participants, disease and intervention. It is also important that a scale is sensitive to change, otherwise a negative finding could be an artefact of the insensitivity of the scale and not of the real effect of the treatment on QoL. It may be more ethical not to measure QoL at all than to use an inappropriate scale which could give misleading results.

### Quality of reported data

In order for readers to assess the importance of RCT findings, it is essential to have adequate descriptions of the outcomes and data. CONSORT recommends: 'Authors should give full details of how the primary and secondary outcomes were measured' and 'for each primary and secondary outcome, a summary of results for each group and the estimated effect size and its precision (e.g. 95% confidence interval)'.

#### Description of scale and scoring methods

Some papers gave neither a full description of the scale nor an explanation of the scoring method [17,19,30,36,37,39]. Several studies included children but it is not always clear whether the child, parent or a combination responded. Irrespective of the difficulties encountered with the interpretation of a combination of patient and patient proxy data, it is useful to know from whom the data have been obtained. A comprehensive description of the scale and scoring methods is particularly important in cases where scales have been merged across child and adult versions [19,37] or the scale transformed [38]. One paper stated that two scales with different numbers of items and domains had been combined but did not justify this or describe how it had been done [37]. While the statistical power is likely to be increased by such pooling, there is no guarantee that it is clinically meaningful.

#### Baseline data

QoL outcomes are often secondary, and a RCT may not have been powered on them. It is therefore important that adequate summary information is provided. A table of baseline characteristics, by treatment group, allows readers to judge the success of the randomisation. There may be clinically important baseline differences between the groups, or ceiling or floor effects. In several papers it was impossible to assess the validity of the conclusions, as there was incomplete summary information about QoL baseline values [17,19,20,28,34,37].

#### Statistical versus clinical significance

It is important to distinguish between statistical significance and clinical significance. This is addressed in the CONSORT statement as follows: 'The difference between statistical significance and clinical importance should always be borne in mind. Authors should particularly avoid the common error of interpreting a non-significant result as indicating equivalence of interventions. The confidence interval provides valuable insight into whether the trial result is compatible with a clinically important effect, regardless of the p-value'. Most statistical tests will give a confidence interval for the difference, as well as a p-value. A 95% confidence interval gives a range of values, which we can be 95% sure contains the true difference. When the p-value is greater than 0.05, and the 95% confidence interval contains no values of any clinical importance, it is reasonable to conclude that any real difference between treatments is too small to be of any interest. If a study is too small then the observed difference may be too small to reach statistical significance, even in the presence of a genuine difference in treatments. Conversely, in large studies, small changes in both FEV_1 _and QoL scores may be statistically significant as determined by a p-value but the observed differences may be too small to be clinically relevant. In this review there was a tendency for QoL results to be interpreted in terms of statistical significance. Of the two papers where QoL was the main outcome, Quittner [14] provided a full description of the data, whereas Wolter [20] gave no confidence intervals or measures of variability. Only three studies in which QoL was a secondary outcome reported confidence intervals [19,24,31] even though the studies were not powered on the QoL outcome. Most reported standard deviations or standard errors but three gave no description of variability [17,27,39]. Overall, there was little or no discussion of QoL results when they were statistically non-significant. Neither was there adequate discussion of the relationship between QoL results and those for the primary outcomes.

### Types of trials, sample size and QoL

#### Superiority trials

It is important that an RCT has adequate power. The majority of RCTs are superiority trials, where the aim is to test whether one treatment is better than another (or than placebo). CONSORT states: 'Ideally a study should be large enough to have a high probability (power) of detecting a statistically significant or clinically important difference of a given size if such a difference exists'... 'Reports of studies with small samples frequently include the erroneous conclusion that the intervention groups do not differ, when too few patients were studied to make such a claim'. In this review some papers did not report a formal sample size calculation for any outcome [24,28,38,39]. One study [24] reported a retrospective power calculation based on the observed data, which is of little value [8]. No study had an a priori sample size calculation for QoL when this was a secondary outcome.

#### Crossover trials

In some circumstances using a crossover design can alleviate the problem of requiring a large sample. Four crossover trials were located [17,24,31,39]. Crossover trials are useful where the effect of a treatment can be switched on and off, and it is ethical to do this. When feasible they can be more efficient than parallel-group designs because each participant acts as his/her own control and a smaller sample is required for the same statistical power. However, although a great deal might be known about the pharmacological effect of a drug in the body and its consequent effect on lung function, much less is understood about possible carryover effects on QoL. This is potentially compounded in studies where there are more than two treatment periods.

#### Non-inferiority trials

There are instances in which the aim is not to demonstrate superiority of one treatment over another, but to show therapeutic equivalence i.e. that one treatment is not inferior to another. The methods used for superiority trials are then of no use; failure to find a statistically significant difference is not the same as establishing equivalence. In fact, it is impossible to show 'exact' equivalence, so researchers have to define the range of values, near zero, that are of no clinical interest. This value of 'no difference' must, logically, be less than the minimal clinically important difference (MCID). For example, if the MCID for a QoL scale is 10 points a difference of 3 units between groups would probably be classed as 'equivalent'; it may be less clear how to view a difference of 7 units. This is a clinical decision, not a statistical one, and the values need to be established before the sample size calculation is done. For the two cases in this review where equivalence designs were apparently employed [20,39] there was either a lack of precision in the aim of the trial, or an inappropriate analysis. In the one trial where QoL was the primary outcome [20], the sample size calculation was not presented in terms of equivalence, and was revised after data were collected. This resulted in a retrospective adjustment to the definition of what had been deemed to be a clinically important difference. There was no sample size calculation in the other study [39].

### Missing values

Many RCTs suffer problems with missing values. These can be sporadic (e.g. where a patient misses an appointment or omits to return one questionnaire) or because of dropout, where data are completely missing after a certain time. This can be a common problem in QoL measurement and researchers have to decide how to deal with incomplete data. One way is to omit cases with missing values. If values are missing 'completely at random', then analysis on the complete data can be valid, although it is against the principle of 'intention to treat'. However, missingness is more often related to a change in condition [45]. In practice it is very difficult to be sure that values are missing completely at random, simply because the values are unknown.

The implications of missing data are potentially serious if about 15% or more of the values are missing, or if there are different patterns of missingness between treatment groups. When there is a significant amount of missing data it is highly desirable to check the sensitivity of the results by using more than one statistical method. There are a variety of methods that can handle incomplete data. Different methods give potentially different results, because they make different assumptions about the reasons for missingness. Some methods are relatively simple (e.g. use the last recorded value) but p-values and confidence intervals should really be adjusted. Methods like t-tests and repeated measures ANOVA require complete data. However, there are more sophisticated methods like Generalised Estimating Equations (GEEs) that can be used to analyse longitudinal data with different numbers of observations per participant. Nonetheless, the fact that the analysis is possible does not mean that it is valid. Researchers should compare participants with and without missing data to check for potential systematic differences. Most papers reported missing observations for the QoL outcomes, some of which were substantial [18,31,38]. Only Suri [31] gave any description of dropouts and checked that they were not the most severely affected participants. Orenstein [38] omitted dropouts from the analysis because of potential bias, but did not explore the nature of the dropouts and the possible bias resulting from their exclusion. Some researchers used GEEs for their analysis [18,31,38] but gave incomplete details of the chosen options.

## Conclusion

Authors tend to provide definite statements about QoL, but no trial in this review provided conclusive data. Even well-designed clinical trials can be difficult to manage, and high-quality reporting is essential. In the papers in this review the treatments and patient characteristics were generally well described. Difficulties arose when QoL scales were not described and summary information was not provided. Journal editors place word restrictions on papers so full descriptions of all secondary outcomes are not always possible. However, interpretation was further confounded when the emphasis was purely based on statistical significance ignoring clinical relevance. Although the CONSORT statement is an excellent guide for RCTs, it was not available when some of the trials were reported, and it does not specifically address QoL assessment and psychometric validity [46]. Properly validated disease-specific QoL measures are most appropriate for the evaluation of CF therapies as they include the relevant items that are likely to be sensitive to change [47]. Without suitable guidelines it is difficult for authors to employ QoL scales appropriately. This review highlights many of the pitfalls of QoL measurement in CF clinical trials and provides constructive information concerning QoL in trial design and the reporting of QoL data.

## Authors' contributions

Both authors planned the study, independently located and reviewed the trials, drafted the paper and agreed the final version
